# Modulation of immune response via cytokine gene expression and cecal microbiota in rabbits: Changes caused by dietary supplementation of *Arthrospira platensis* and *Chlorella vulgaris*

**DOI:** 10.5455/javar.2026.m1015

**Published:** 2026-03-09

**Authors:** Ahmed A. A. Khattab, Mohammed F. El Basuini, Safaa E. S. Atia, Nabila E. M. El-Kassas, Othman Y. Alyahyawy, Amera F.M. Zaitoun, Salma H. Abu Hafsa

**Affiliations:** 1 Faculty of Agriculture, Tanta University, Tanta 31527, Egypt; 2 King Salman International University, South Sinai 46612, Egypt; 3 Animal Production Research Institute, Agricultural Research Center, Dokki, Giza, Egypt; 4 Department of Medical Laboratory Technology, Faculty of Applied Medical Sciences, King Abdulaziz University, Rabigh, Saudi Arabia; 5 Department of Agricultural Botany, Faculty of Agriculture Saba Basha, Alexandria University, Alexandria, Egypt; 6 Livestock Research Department, Arid Lands Cultivation Research Institute, City of Scientific Research and Technological Applications, New Borg El-Arab 21934, Alexandria, Egypt

**Keywords:** Performance, immune response, gene expression, *Spirulina platensis*, *Chlorella vulgaris*, cecal microbiota, rabbits

## Abstract

**Objectives:** This study was aimed to evaluate the effects of dietary supplementation with the microalgae *Arthrospira platensis* (Ap) and *Chlorella vulgaris* (Cv) on growth performance, oxidative stress, immune-related cytokines gene expression, cecal fermentation, microbial population, and protein profile changes in growing rabbits.

**Materials and Methods:** Seventy-five male rabbits aged five-week were randomly allocated to five groups (*n* = 15). The control group received a basal diet without supplementation, whereas the remaining four groups were received basal diets supplemented with Ap or Cv at levels of 300 mg/kg or 500 mg/kg diet for 56 days.

**Results:** Phytochemical analysis revealed that both microalgae were rich in bioactive compounds including flavonoids, terpenoids and tannins exhibiting strong antioxidant activity. Dietary supplementation with Ap and Cv significantly improved BWG and FCR, while decreasing feed consumption. Oxidative stress markers, including malondialdehyde and hydrogen peroxide, were significantly decreased in algal-supplemented groups, accompanied by enhanced antioxidant status. Immune responses were favorably modulated through the upregulation of anti-inflammatory cytokines, particularly (*IL-10* and *IL-4*). In addition, dose-dependent changes in protein expression profiles were observed in serum, liver and spleen tissues. Cecal fermentation parameters were improved, as evidenced by increased total volatile fatty acids, acetic and propionic acids, as well as *Lactobacillus* spp., along with reduced NH_3_-N and pathogenic bacterial populations of *E. coli, Staphylococcus* spp., *Enterococcus* spp., and total coliforms (*p* < 0.05).

**Conclusions:** Dietary supplementation with *A. platensis* and *C. vulgaris* improves growth performance, antioxidant capacity, immune function, and cecal health in growing rabbits, supporting their use as feed additives.

## 1. Introduction

Rabbits are increasingly recognized as sustainable sources of meat production due to their excellent feed efficiency, rapid growth rates, and adaptability to a range of farming conditions [1]. Rabbits exhibit superior efficiency in converting feed into high-quality animal protein compared with other livestock species. Rabbit meat is highly nutritious, characterized by a high protein content (~25%), low fat and cholesterol (~4%), and a moderate calorie value (160 kcal/100 gm) [2]. Despite these advantages, rabbit production faces significant challenges, such as susceptibility to gastrointestinal disorders, poor nutrient utilization, and growing global restrictions on the use of antibiotics in animal and poultry farming [3]. To enhance animal health, growth, reproductive efficiency, and overall productivity, feed supplements are commonly incorporated into animal diets [4, 5]. In this context, microalgae-derived supplements, specifically *Chlorella vulgaris* (Cv) and *Arthrospira platensis* (Ap), are gaining popularity due to their high nutrient density and broad spectrum of biological activities. These microalgae possess antioxidant, immunomodulatory, and antimicrobial properties, and are considered promising alternatives to conventional feed ingredients [6, 7, 8]. These microorganisms are natural sources of high-quality proteins, polyunsaturated fatty acids, pigments, vitamins, and bioactive compounds that modulate gut health, immunity, offering dual health benefits without contributing to antimicrobial resistance [7, 9, 10]. *A. platensis*, a blue-green microalgae, is particularly rich in essential nutrients, including proteins, vitamins, minerals, and potent antioxidants, such as phycocyanin, phenolic compounds, and flavonoids [10, 11, 12]. These bioactive compounds have been associated with a wide range of therapeutic effects, including immune modulation and anti-inflammatory properties, and the mitigation of oxidative stress [10]. Beyond their nutritional value, these compounds have demonstrated antiviral potential alongside their anti-inflammatory properties [13].

Similarly, *C. vulgaris* has gained considerable interest as a valuable protein source for both human and animal nutrition due to its diverse biochemical composition [14]. It is rich in minerals, vitamins, and bioactive molecules, including polysaccharides, carotenoids, and chlorophyll, which promote cellular defense mechanisms by modulating oxidative stress and stimulating immune responses, including increased glutathione activity in rabbits fed *C. vulgaris* [15, 16]. Furthermore, its high protein content also makes it an effective alternative to protein sources such as soybean meal. Numerous studies have confirmed the biofunctional properties of both *C. vulgaris* and *A. platensis*, demonstrating their antioxidant, anti-inflammatory, and antimicrobial properties [14, 17, 18]. These microalgae have been shown to suppress the synthesis of key pro-inflammatory cytokines, including *TNF-α, IL-1β*, and *IL-6* [18, 19, 20, 21]. For example, phycocyanin from *A. platensis* exhibits strong neuroprotective and anti-inflammatory impacts, while chlorophyll and carotenoids from *C. vulgaris* support detoxification processes and antioxidant defense mechanisms. Despite the growing body of evidence supporting the health-promoting properties of *A. platensis* and *C. vulgaris*, data regarding their effects on oxidative stress biomarkers, immune-related cytokine gene expression, and serum and tissue protein profiles in rabbits remain limited.

Therefore, it was hypothesized that dietary supplementation with *A. platensis* and *C. vulgaris* would exert beneficial effects on rabbit health and performance. Accordingly, the present study aimed to investigate the effects of dietary inclusion of A. platensis and C. vulgaris on growth performance, antioxidant capacity, immune modulation via cytokine gene expression, alterations in serum and tissue protein profiles, and cecal fermentation characteristics and microbial populations in rabbits.

## 2. Materials and Methods

### 2.1. Ethical approval

The experiment was performed at a private farm under the supervision of the Animal Production Department, Faculty of Agriculture, Tanta University, and Arid Lands Cultivation Institute, SRTA-CITY, New Borg El-Arab, Alexandria, Egypt. All experimental procedures were approved by the Ethical Committee of the Faculty of Agriculture, Tanta University, for the care and use of animals in research (protocol No. AY2019-2020).

### 2.2. Cultivation optimization of microalgae

The cyanobacterium *A. platensis* and the green microalga *C. vulgaris* were cultivated under controlled laboratory conditions. The purified isolate of *A. platensis* was grown in 1000 ml flasks containing sterilized Zarrouk medium, whereas *C. vulgaris* was cultured in 1000 ml flasks containing sterilized Bold’s Basal Medium (BBM) adjusted to a final pH of 6.3. Each culture was inoculated with 10% (v/v) an exponentially growing starter culture standardized to an optical density of approximately 0.6 at 680 nm. Cultures were incubated at 25 ± 2°C under a 16:8 h light–dark photoperiod using cool white, fluorescent lamps, providing a light intensity of approximately 1.2 Lux at the culture surface. Continuous agitation at 130 rpm was maintained using an orbital shaker to ensure adequate mixing and gas exchange. Cultures were monitored daily, and biomass was harvested during the exponential growth phase (days 7–10). Biomass harvesting was performed by centrifugation at 5,000 rpm for 10 min. The resulting pellets were washed twice with sterile distilled water to remove residual culture medium and salts, then dried in a hot-air oven (Binder, Germany) at 60°C for 12 h until constant weight. The dry biomass yield was approximately 1.8 gm/l for *A. platensis* and 0.4 gm/l for *C. vulgaris*. The dried biomass was stored in airtight containers at room temperature until further analysis.

### 2.3. Proximate analysis of algae biomass

The proximate composition of dried *A. platensis* or *C. vulgaris* biomass was determined according to standard AOAC methods [22] (Table 1). Dried samples of *A. platensis* and *C. vulgaris* were finely ground using a Cyclotec mill equipped with a 1-mm screen (Cyclotec 1093; Foss, Germany) and stored until chemical analysis. Moisture content was determined by drying approximately 2 gm of sample in an oven at 70°C until a constant weight. Crude protein content was determined using Kjeldahl’s method (AOAC method no. 978.04), applying a nitrogen conversion factor of 6.25. Crude fat content was determined by the Soxhlet extraction method using petroleum ether (40 – 60°C) (AOAC method no. 930.09). Ash content was determined by incinerating samples in a muffle furnace at 600–625°C following the AOAC method 942.05. Total fiber content was determined following the AOAC method 991.43. Total carbohydrate content was calculated by difference.

**Table 1.** Proximate analysis of *A. platensis* and *C. vulgaris* algal biomass (% on a DM basis).

Parameters	*C. vulgaris*	*A. platensis*
Dry matter	90.39	91.20
Protein	43.6	56.4
Lipids	20.19	6.6
Carbohydrates	23.8	26.2
Fiber	8.80	4.10
Nitrogen-Free Extract	39.69	53.90
Ash	13.56	12.3

### 2.4. Phytochemical profile of algae extracts

Qualitative phytochemical screening of *A. platensis* and *C. vulgaris* extracts was performed according to the standard protocols described by Blažeković et al. [23] and Iqbal et al. [24]. Extracts at 10 mg/ml were analyzed for the presence of steroids, saponins, phenols, tannins, flavonoids, and terpenoids by characteristic color changes or precipitate formation. All tests were performed in triplicate.

### 2.5. Total phenolic content of Algae extracts

The total polyphenolic content (TPC) of *A. platensis* and *C. vulgaris* extracts was determined using the Folin-Ciocalteu colorimetric method as described by Jain et al. [25]. Briefly, 0.3 ml of each algal extract was mixed with 75 µl of Folin-Ciocalteu reagent and 5 ml of distilled water, then incubated at 25°C for 30 min. Subsequently, 0.5 ml of 7.5% (w/v) sodium carbonate (Na₂CO₃) was added, and the final volume was adjusted with distilled water. The reaction mixture was incubated in the dark at room temperature for 90 min. A gallic acid standard curve (25, 50, 75, 100, 125, and 150 µg/ml) was prepared for calibration (R^2^ > 0.99). Absorbance was measured at 760 nm using a UV-Vis Spectrophotometer (Jenway, Japan) against a reagent blank. Total phenolic content was calculated from the standard curve and expressed as mg gallic acid equivalents per gram of dry weight (mg GAE/gm DW).

### 2.6. Total flavonoid content of algal extracts

The total flavonoid content (TFC) of *A. platensis* and *C. vulgaris* extracts was determined using the aluminum chloride colorimetric method as described by Chang et al. [26]. Briefly, 200 μl of algal extract was mixed with 1.0 ml of methanol, 0.5 ml of distilled water, 50 μl of 10% aluminum chloride, and 50 μl of 1 M potassium acetate. The mixture was incubated in the dark at room temperature for 30 min. Absorbance was measured at 415 nm using a UV-Vis spectrophotometer (Jenway, Japan). Total flavonoid content was calculated from a quercetin standard curve and expressed as milligrams of quercetin equivalents per gram of dry weight (mg QE/gm DW), where quercetin was used as a standard.

### 2.7. Antioxidant activity of algal extracts

The antioxidant activity of *A. platensis* and *C. vulgaris* extract was evaluated using 2,2-diphenyl-1-picrylhydrazyl (DPPH) and 2,2′-azinobis-(3-ethylbenzothiazoline-6-sulfonic acid) (ABTS) radical scavenging assays. Algal extracts were dissolved in 1.0% dimethyl sulfoxide (DMSO) and tested at concentrations of 50, 100, and 150 µg/ml. For the DPPH assay, a freshly prepared 0.1 mM DPPH solution was mixed with each extract and incubated in the dark at room temperature for 30 min. Absorbance was measured at 517 nm using a UV-Vis spectrophotometer (Jenway, Japan). For the ABTS assay, the ABTS radical cation (ABTS•^+^) was generated by reacting ABTS with potassium persulfate and subsequently diluted with distilled water to an absorbance of 0.70 ± 0.02 at 734 nm prior to use, according to the method described by Zengin et al. [27]. Radical scavenging activity was calculated, and IC₅₀ values were determined using nonlinear regression analysis.

### 2.8. Animals and management

A total of 75 growing male New Zealand White (NZW) rabbits, aged 35 days old and with an initial body weight of 665.93 ± 15.18 gm, were randomly divided into five experimental groups in a completely randomized experimental design. Each group consisted of three replicates, with five rabbits per replicate. The experimental microalgae, *A. platensis* (Ap) and *C. vulgaris* (Cv) were chemically analyzed to evaluate their nutrient composition and to formulate experimental diets (Table 1). After drying, the algal biomass was finely ground and incorporated into the basal diet at the experimental inclusion levels. Diets were subsequently pelleted to ensure uniform distribution and to maintain the stability and biological activity of the supplements. The five dietary groups were as follows: a control group fed a basal diet without supplementation; two groups supplemented with *A. platensis* at 300 mg/kg (Ap1) and 500 mg/kg (Ap2) diet; and two groups supplemented with *C. vulgaris* at 300 mg/kg (Cv1) and 500 mg/kg (Cv2) diet. All experimental diets were formulated to meet the nutritional requirements of growing rabbits as recommended by De Blas and Mateos [28] (Table 2). Rabbits were housed individually in galvanized wire battery cages (50 cm × 40 cm × 35 cm) in a well-ventilated building under standardized management conditions. Environmental conditions were maintained at an ambient temperature of 23°C ± 2°C, relative humidity of 55%–65% and a 12 h light/dark cycle. Feed and fresh water were provided *ad libitum* throughout the 56-day experimental period. All diets were free of antibiotics.

**Table 2.** Ingredients composition and chemical analysis of the basal diet (% as dry matter basis).

Feed ingrediants	%
Yellow corn	17.6
Soybean meal (44%)	16.3
Berseem hay	29.14
Barley grain	12.7
Wheat bran	18.2
Molasses	2.3
Limestone	1.3
Dicalcium Phosphate	0.7
Salt	1.5
Premix*	0.2
DL-Methionine	0.06
Total	100
Calculated analysis	
Crude Protein	19.1
ether extract	3.1
Crude Fat	14.4
Ash	3.2
DL-Methionine	0.07
Lysine	0.95
Calcium	0.88
Total phosphorus	0.52
Digestible energy (kcal/kg)	2743 kcal/kg

*Premix provided each kg of feed with Biotin 0.05 mg; Choline = 250 mg; Zn = 50 mg; Se = 0.1 mg; Fe = 50 mg; Cu = 5 mg; Co = 0.1 mg; Folic acid = 3 mg; I = 0.2 mg; Mn = 85 mg; Niacin = 50 mg; Pantothenic acid = 10 mg; Vitamin A = 6000 IU; Vitamin D3 = 900 IU; Vitamin E = 40 mg; Vitamin K3 = 2 mg; Vitamin B1 = 2 mg; Vitamin B2 = 4 mg; Vitamin B6 = 2 mg; Vitamin B12 = 0.01 mg. DM, Dry matter; CP, Crude protein; EE, Ether extract; CF, Crude fiber.

### 2.9. Growth performance measurements

Rabbits were weighed weekly throughout the experimental period to monitor body weight changes. Average daily weight gain, feed consumption (FC, gm/head/day), and feed conversion ratio (FCR; gm feed /gm weight gain) were calculated accordingly. The performance index (PI%) was calculated based on the live body weight and FCR as follows:


1 \[
{\mathrm{Performance index}}\left({{\mathrm{PI}}\% } \right) = \left[ {{\mathrm{live body weight}}\;\left({{\mathrm{kg}}} \right){\mathrm{/FCR}}} \right] \times {\mathrm{1}}00
\]

### 2.10. Cecal microbiota and fermentation patterns

At the end of the experiment, ten rabbits from each group were euthanized to evaluate cecal fermentation characteristics and microbial populations. Following careful excision of the cecum, its contents were gently expressed, strained through two layers of sterile gauze, and the pH was immediately measured using a digital pH meter (GLP 21 model; CRISON, Barcelona, Spain).

The strained cecal fluid was centrifuged at 7000×*g* for 10 min at 20°C, and the supernatant was divided into two aliquots. One aliquot was preserved with 5% (v/v) orthophosphoric acid and 1% (w/v) mercuric chloride (0.1 ml/ml sample) for the determination of total and individual VFAs proportions. Volatile fatty acids, including acetic, propionic, and butyric acids, were quantified by high-performance liquid chromatography (HPLC; Model Waters 600; UV detector, Millipore Corp., Burlington, MA, USA) according to previously described methods [29, 30].

VFA concentrations were expressed in mmol/l. The second aliquot was acidified with 0.2 M hydrochloric acid (1 ml/ml sample) for the determination of ammonia nitrogen (NH₃–N) concentration using spectrophotometric analysis [31]. For microbial analysis, fresh cecal contents were collected aseptically into sterile tubes. Tenfold serial dilutions were prepared in phosphate-buffered saline and plated onto appropriate selective media. Total bacteria, *Lactobacillus* spp., and *Enterococcus* spp. were cultured on de Man Rogosa-Sharpe (MRS) agar and incubated anaerobically at 37°C for 48 h. While *E. coli, Staphylococcus* spp., and total coliforms were cultured on MacConkey agar and incubated aerobically at 37°C for 24 h. Bacterial colony-forming units (CFU) were counted in Petri dishes, with counts ranging from 30–300 CFU/gm, depending on the growth characteristics of the bacterial species. The counts were expressed as log CFU/gm.

### 2.11. Sampling procedure

At the end of the experiment, five rabbits from each group were slaughtered to collect blood and tissue samples. Three blood samples (5 ml each) were collected into clean tubes without anticoagulants for serum separation. The samples were centrifuged at 3,000 rpm for 10 min. After which the serum was carefully transferred into Eppendorf tubes and stored at –20ºC for further analysis. Serum malondialdehyde (MDA) levels were determined using commercial kits (Biodiagnostic Company Giza, Egypt) according to the manufacturer’s instructions. Liver and spleen tissues were immediately separated on ice, rinsed with 0.90% saline solution (pH 7.5), and rapidly frozen in liquid N₂ for subsequent analyses.

### 2.12. Oxidative stress assay

Serum MDA levels were quantified using the thiobarbituric acid reactive substances (TBARS) assay, measured spectrophotometrically at 532 nm, as described by Ohkawa et al. [32]. Hydrogen peroxide (H₂O₃) levels were determined using a commercial hydrogen peroxide assay kit, with absorbance measured at 570 nm. Both MDA and H₂O₂ concentrations were expressed as nmol/ml [33].

### 2.13. Cytokine gene expression

#### 2.13.1. Total RNA extraction and cDNA synthesis

Total RNA was extracted from serum, spleen, and liver samples using TRIzol-based RNA extraction kit (Total RNA Purification Mini Spin Kit), according to the manufacturer’s instructions. The quality and quantity of the extracted RNA were determined using a BioDrop spectrophotometer (BioDrop μLite, UK). RNA samples with an optical density (O.D.) ratio of 1.8–2.0 were considered sufficiently pure and selected for subsequent analyses. To eliminate genomic DNA contamination, total RNA was treated with DNase I using the RNeasy Mini Kit. RNA integrity was verified by electrophoresis on a 1.5% denaturing agarose gel prior to cDNA synthesis. Complementary DNA (cDNA) was synthesized from total RNA using the ProtoScript First Strand cDNA Synthesis Kit (Biolab, UK) according to the manufacturer’s protocol. The synthesized cDNA was initially verified by conventional PCR and then stored at – 80°C until further use in qRT-PCR analysis.

#### 2.13.2. Quantitative real-time PCR (qRT-PCR)

Quantitative real-time PCR was performed to amplify both target and reference genes using Light Cycler^®^ 480 system (Roche, Germany) with an EvaGreen qPCR reaction kit (Qarta). It was carried out using the SYBR Green following the manufacturer’s instructions. Primer pairs were designed using primer 3 software based on sequences retrieved from the NCBI database and the Ensembl genome browser (EMBLEBL Wellcome Trust Sanger Institute, Cambridgeshire, UK) (Table 3). The qRT-PCR cycling conditions were as follows: initial denaturation at 95°C for 15 min; 40 cycles at 94°C for 15 sec; annealing at 56°C for 30 sec, and extension at 72°C for 30 sec. Expression of GAPDH was used as an endogenous reference gene. The expression levels of cytokine genes, including *IL-2, IL-4, IL-6, IL-10, IL-2*2, *TNF-α*, and *IFN-γ*, were evaluated in all samples. Relative gene expression was calculated using 2^ (–ΔΔCt) method [34].

**Table 3.** Oligonucleotide primer sequences used for quantitative RT-PCR analysis.

Gene Name		Sequence	Product size (bp)	NCBI Accession
*IL-2*	F	5′-TGA AAC ATC TTC AGT GTC TAG AAG-3′	128	Z36904
	R	5′-CAT TGT AGA ATT TCT GAA CAG AT-3′		
*IL-4*	F	5′-GTC ACT CTG CTC TGC CTC CTC-3′	121	AF169170
	R	5′-GGA CTC GAC AGG AAC CTC TG-3′		
*L-6*	F	5′-CTA CCG CTT TCC CCA CTT CAG-3′	143	NM_001082064
	R	5′-TCC TCA GCT CCT TGA TGG TCT C-3′		
*IL-10*	F	5′-GAG AAC CAC AGT CCA GCC AT-3′	110	D84217
	R	5′-CAT GGC TTT GTA GAC GCC TT-3′		
*IL-2*2	F	5′-ACC TCA CCT TCA TGC TGG CTA A-3′	137	XM_002711248
	R	5′-CAT GGA ACA GCT CAT TCC CAA T-3′		
*TNF-α*	F	5′-GTC TTC CTC TCT CAC GCA CC-3′	115	M12845
	R	5′-TGG GCT AGA GGC TTG TCA CT-3′		
*IFN-γ*	F	5′-TTC TTC AGC CTC ACT CTC TCC-3′	134	D84216
	R	5′-TGT TGT CAC TCT CCT CTT TCC-3′		
*GAPDH*	F	5′-TGA CGA CAT CAA GAA GGT GGT G-3′	143	NM_001082253
	R	5′-GAA GGT GGA GGA GTG GGT GTC-3′		

### 2.14. Soluble protein pattern changes

Soluble protein profiles among the experimental animal groups were analyzed using sodium dodecyl sulfate-polyacrylamide gel electrophoresis (SDS-PAGE) as described by Mohsin et al. [35]. Spleen and liver tissues (100 mg each) and serum (50 μl) were homogenized in lysis buffer containing 2% SDS and protease inhibitors for 2 h, followed by centrifugation at 12000 rpm for 10 min. Aliquots of 10 μl from the supernatant were resolved on a 12% SDS-PAGE gel at 100 V for 90 min. Gels were stained with Coomassie Brilliant Blue R-250, and excess dye was removed by glacial acetic acid. Protein bands were visualized and digitized using Gel Doc XR+ imaging software. Molecular weights (10–250 kDa) and band intensities were quantified using ImageJ software. Dendrograms were generated using the unweighted pair group method with arithmetic mean (UPGMA) and Jaccard’s similarity coefficient in NTSYS-pc to assess similarities among protein profiles across experimental groups. Protein banding in serum, spleen, and liver samples was compared to those of the control group to identify changes in molecular weight distribution and band intensity.

### 2.15. Statistical analysis

The experimental data were statistically analyzed using analysis of variance within the general linear model of SPSS software (v. 16.0, SPSS Inc., Chicago, IL) according to the following statistical model:


2 \[
{{\mathrm{Y}}_{{\mathrm{ij}}}} = {\mathrm{\mu }} + {{\mathrm{T}}_{\mathrm{i}}} + {\varepsilon _{{\mathrm{ij}}}}
\]

where Y_ij_ presents the observed value, μ is the overall mean effect, T_i_ is the i^th^ treatment effect, and ε_ij_ is the random error associated with the jth rabbit in the i^th^ treatment group. Differences among means were considered statistically significant at *p* < 0.05. All results are expressed as least-squares means. A heat map was generated by converting qRT-PCR data into relative gene expression values to visually represent expression differences among groups. Hierarchical clustering was conducted based on average linkage method and Euclidean distance metrics.

## 3. Results

### 3.1. Proximate analysis of algal biomass

The proximate composition of *A. platensis* and *C. vulgaris* biomass used as dietary supplements in rabbit diets is presented in Table 1. The results showed that *A. platensis* biomass contained higher contents of crude protein (56.4%) and carbohydrate (26.2%), whereas *C. vulgaris* showed a higher lipid content (20.19%). The contents of crude protein, carbohydrate, and lipid in *C. vulgaris* were 43.6 %, 23.8 % and 20.19 %, respectively. Notable variations in ash and fiber contents were also observed between the two algal biomasses, as shown in Table 1.

### 3.2. Phytochemical profile of algal extracts

Qualitative Phytochemical screening of methanolic extracts from *A. platensis* and *C. vulgaris* revealed the presence of several classes of secondary metabolites. The *A. platensis* extract showed high levels of phenolic compounds, terpenoids, and steroids, along with moderate amounts of flavonoids, tannins, and saponins. In contrast, phytochemical screening of *C. vulgaris* extracts revealed a rich composition of bioactive compounds, characterized by high levels of phenolics and flavonoids, moderate amounts of terpenoids, and minor amounts of tannins and saponins; however, steroids were not detected.

### 3.3. Total phenolic and flavonoid contents of algal extracts

Quantitative determination of total phenolic content (TPC) and total flavonoid content (TFC) is shown in Table 4. The methanolic extract of *A. platensis* exhibited a higher TPC (29.15 mg GAE/gm DM) compared to *C. vulgaris* (16.82 mg GAE/gm DM). Conversely, *C. vulgaris* showed a slightly higher rate of TPC (9.30 mg QE/gm DM) than *A. platensis* (8.48 mg QE/gm DM).

**Table 4.** Antioxidant properties of *A. platensis* and *C. vulgaris* extracts, including DPPH and ABTS radicals scavenging activity and total phenolic and flavonoid contents.

Items	*A. platensis*	*C. vulgaris*	Ascorbic acid*	SEM	*p* value
DPPH (IC₅₀, μg/ml)	187^b^	192.3^a^	75.4^c^	2.345	0.002
ABTS (IC₅₀, μg/ml)	234.2^a^	251.7^b^	82.6^c^	1.17	0.003
TPC (mg GAE/gm DM)	29.15^a^	16.82^b^	–	0.64	0.004
TFC (mg QE/gm DW)	8.48^b^	9.30^a^	–	0.42	0.003

Ascorbic acid was used as a positive control for DPPH and ABTS assays. DPPH, 1,1-diphenyl-2-picrylhydrazyl; ABTS, 2,2′-azino-bis(3-ethylbenzothiazoline-6-sulfonate); TPC, total phenolic content; TFC, total flavonoid content.

### 3.4. Antioxidant activity of algal extracts

The DPPH and ABTS radical scavenging assays were used to assess the antioxidant activity of the methanolic algal extracts (Table 4). The IC50 values for the extracts from *A. platensis* (187 μg/ml) and *C. vulgaris* (192.3 μg/ml) in the DPPH assay were substantially higher (*p* < 0.05) than the standard antioxidant ascorbic acid (75.4 μg/ml). In a similar vein, the ABTS radical scavenging assay revealed greater IC50 values (*p* < 0.05) for *A. platensis* (234.2 μg/ml) and *C. vulgaris* (251.7 μg/ml) than for ascorbic acid (82.6 μg/ml), the standard antioxidant.

### 3.5. Growth performance

**Figure 1.**
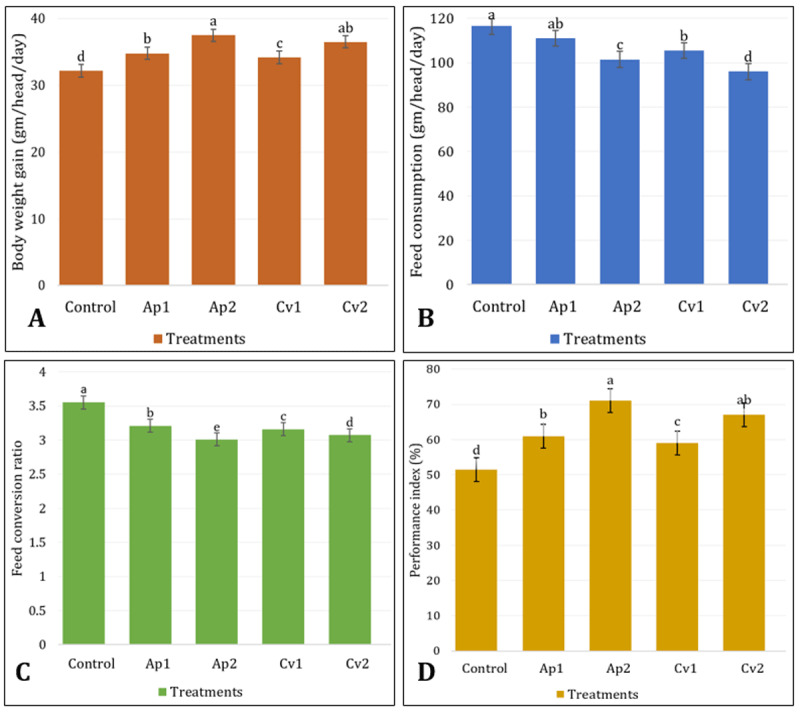
Effect of *A. platensis* and *C. vulgaris* supplementation on growth performance of rabbits: (A) body weight gain, (B) Feed consumption, (C) Feed Conversion Ratio, and (D) Performance index.

The growth performance of rabbits fed the experimental diets over a 56-day period is shown in Figure 1. Rabbits fed diets supplemented with Ap2 and Cv2 exhibited the highest daily body weight gain and performance index PI% (*p* < 0.05), followed by the Ap1 and Cv1 groups, compared to the control group. Compared with the control group, feed consumption was lowest in the Ap2 and Cv2 groups, followed by the Ap1 and Cv1 groups. The best feed conversion ratio (FCR) was observed in the Ap2 and Cv2 groups (*p* < 0.05), followed by the Ap2 and Cv2 groups. Moreover, the rabbits fed Ap1 and Cv1 diets achieved the highest PI% (59.4% and 60.3%, respectively), followed by the Ap1 and Cv1 groups (25% and 38.3%, respectively).

### 3.6. Cecal microbiota and fermentation patterns

The effects of dietary supplementation with two levels of *A. platensis* and *C. vulgaris* on cecal fermentation characteristics and microbiota populations are presented in Table 5. All algal-treated groups exhibited similar cecal pH values, butyric acid concentrations, and total anaerobic bacterial counts. Dietary inclusion of both algae species at the two tested levels resulted in a significant increase in total VFAs, as well as acetic and propionic acid concentrations in cecal contents (*p* < 0.05), while NH₃ – N concentrations was significantly decreased compared to the control group (*p* < 0.05). Regarding microbial populations, counts of total aerobic bacteria, *E. coli, Staphylococcus* spp., *Enterococcus* spp., and total coliform bacteria were significantly decreased in algae-supplemented groups (*p* < 0.05). In contrast, *Lactobacillus* spp. counts in the rabbit’s cecum were dramatically increased (*p* < 0.05).

**Table 5.** Cecal fermentation and microbiota population in rabbits fed algal-supplemented diets.

Items	Control	*A. platensis*		*C. vulgaris*		SEM	*p*-value
		Ap1	Ap2	Cv1	Cv2		
Cecal fermentation patterns							
pH	6.23	6.24	6.21	6.23	6.24	0.03	0.849
Total VFAs (mmol/l)	55.82^c^	60.46^ab^	59.43^b^	61.25^a^	60.35^ab^	0.71	0.004
NH3-N (mmol/l)	12.48^a^	11.74^b^	11.65^b^	11.27^c^	11.42^bc^	0.14	0.012
Acetic acid (mmol/l)	42.60^c^	47.83^ab^	46.93^b^	49.47^a^	47.91^ab^	1.26	0.041
Propionic acid (mmol/l)	19.57^b^	22.21^a^	22.26^a^	22.69^a^	22.41^a^	0.47	0.002
Butyric acid (mmol/l)	6.34	6.41	6.42	6.39	6.41	0.11	0.733
Cecal microbiota counts (log cfu/gm cecal digesta)							
Total anaerobic bacteria	5.21	5.19	5.20	5.15	5.27	0.11	0.844
Total aerobic bacteria	6.51^a^	5.46^b^	5.73^b^	5.48^b^	5.66^b^	0.28	0.027
*Lactobacillus* sp.	6.17^b^	7.43^a^	7.20^a^	7.54^a^	7.29^a^	0.21	0.053
*E. coli*	5.70^a^	3.92^b^	4.33^b^	3.98^b^	4.32^b^	0.37	0.033
*Staphylococcus* sp.	2.66^a^	1.79^b^	1.91^b^	1.86^b^	1.83^b^	0.11	0.015
*Enterococcus* sp.	3.17^a^	1.25^b^	1.19^b^	1.15^b^	1.03^b^	0.25	0.017
Total coliform bacteria	2.78^a^	1.13^b^	1.11^b^	1.07^b^	1.01^b^	0.11	0.011

^a–c^ Means within a row with different superscripts are significantly different (*p* < 0.05). VFA: Volatile fatty acids.

### 3.7. Oxidative stress assay

**Figure 2.**
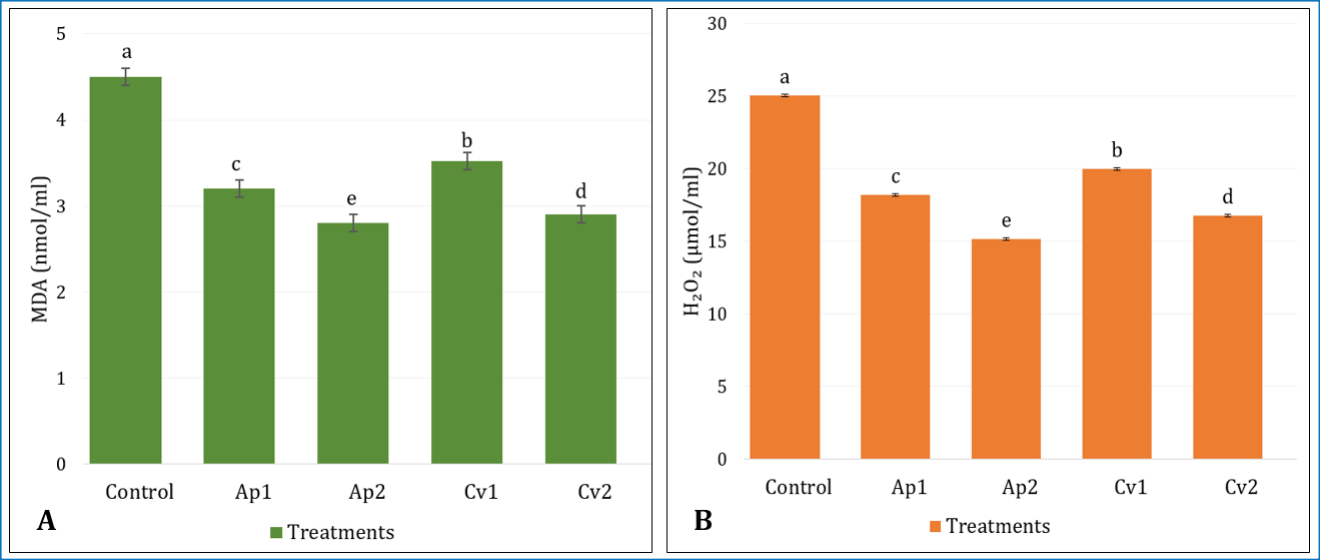
Effects of *A. platensis* and *C. vulgaris* supplementation on serum oxidative stress markers in rabbits: (A) Lipid peroxidation and (B) hydrogen peroxide.

Oxidative stress markers in rabbit serum are illustrated in Figure 2. Serum MDA levels were significantly lower in rabbits fed algae-supplemented diets compared to the control group (*p* < 0.05). The lowest MDA concentrations were observed in Ap2 (2.8 nmol/ml) and Cv2 (2.9 nmol/ml) groups. Similarly, hydrogen peroxide (H₂O₂) levels were considerably lower (*p* < 0.05) in the Ap2 (15.16 μmol/ml) and Cv2 (16.76 μmol/ml) groups compared to the control group (25.04 μmol/ml), indicating a pronounced antioxidant effect of algal supplementation.

### 3.8. Cytokine gene expression

**Figure 3.**
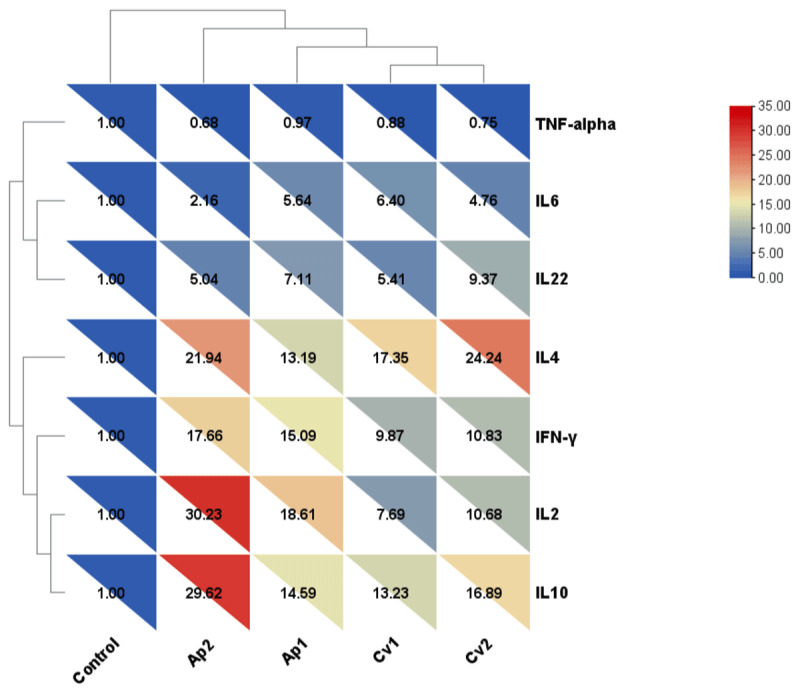
Heatmap and hierarchical clustering of seven differentially expressed cytokines in the serum of rabbits fed diets supplemented with *A. platensis* and *C. vulgaris*.

**Figure 4.**
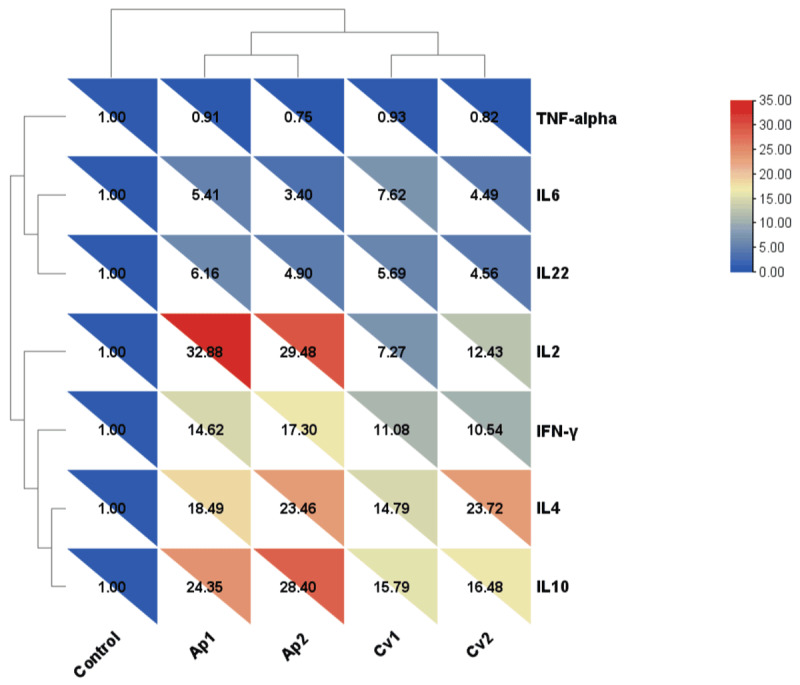
Heatmap and hierarchical clustering of seven differentially expressed cytokines in the liver of rabbits fed diets supplemented with *A. platensis* and *C. vulgaris*.

**Figure 5.**
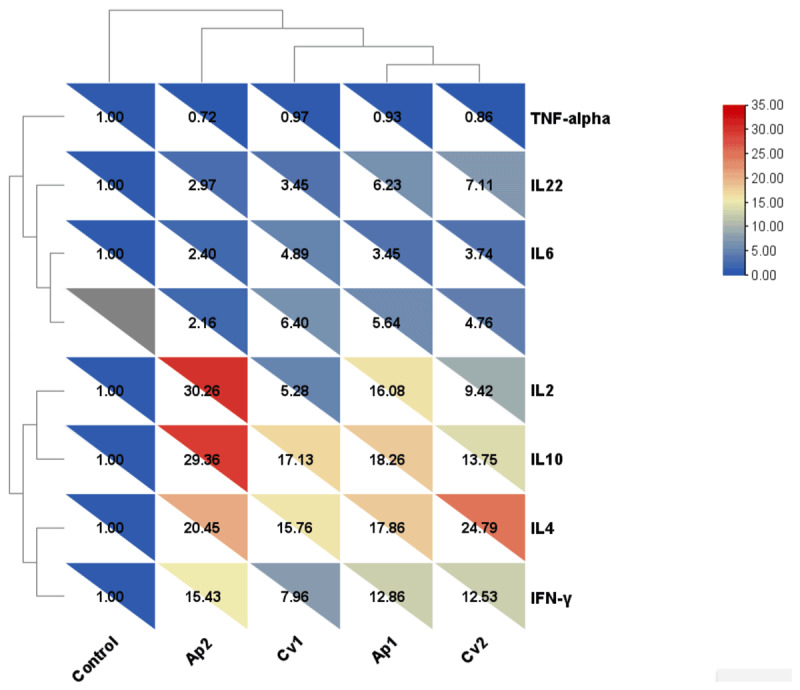
Heatmap and hierarchical clustering of seven differentially expressed cytokines in the spleen of rabbits fed diets supplemented with *A. platensis* and *C. vulgaris*.

The relative mRNA expression levels of cytokine genes in serum, liver, and spleen samples are presented in Figures 3, 4, 5. In serum samples (Figure 3), *IL-2* expression was significantly upregulated in the Ap1 and Ap2 groups compared to the control group (*p* < 0.05). Interleukin-10 (*IL-10*) expression showed the highest increase in Ap2 (29.62-fold) and Cv2 (16.89-fold) groups. Additionally, *IL-4* expressions were significantly increased in the Cv2 (24.24-fold) and Ap2 (21.94-fold) groups (*p* < 0.05). No significant differences in TNF-α expression were observed among treatment groups. In liver tissue (Figure 4), *IL-2* expression was significantly elevated in the Ap1 (32.88-fold), Ap2 (29.48-fold), and Cv2 (13.43-fold) groups compared to the control group (*p* < 0.05). *IL-10* expression was significantly upregulated in all algae-supplemented groups, with the highest expression detected in the Ap2 (28.4-fold). *IL-4* expression was also significantly increased in the Ap2 and Cv2 groups. While, *TNF-α* expression showed moderate but statistically significant increases in the Ap2 and Cv2 groups compared to the control group (*p* < 0.05). In spleen tissue (Figure 5), *IL-2* expression was significantly higher in Ap2 (30.26-fold) and Cv2 (9.42-fold) groups (*p* < 0.05). Moreover, *IL-10* expression was significantly upregulated in all algae-supplemented groups compared to the control group (*p* < 0.05).

### 3.9. Protein profile changes in algae-fed rabbits

**Figure 6.**
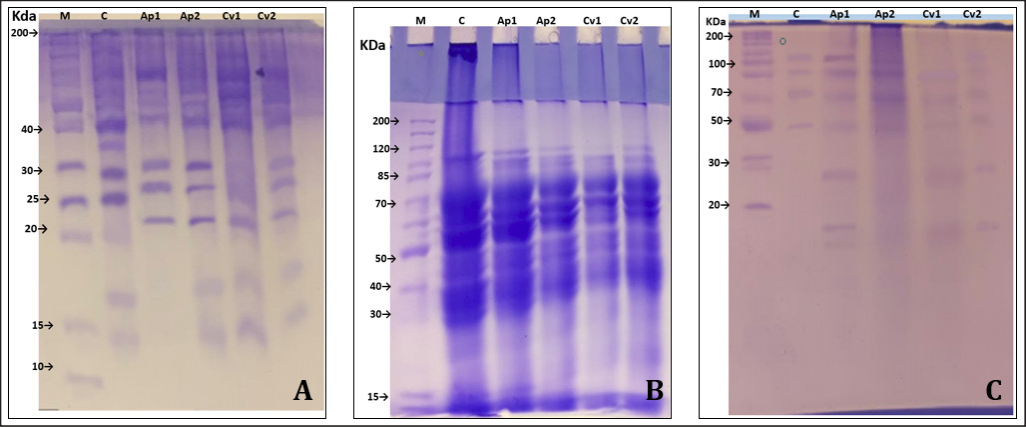
Protein profiles analyzed by SDA-PAGE in serum (A), liver (B), and spleen (C) tissues of rabbits fed diets supplemented with *A. platensis* and *C. vulgaris*.

Protein profile patterns obtained by SDS-PAGE analysis of serum, liver, and spleen samples are shown in Figure 6, along with the corresponding dendrogram cluster analysis in (Table 6). Rabbits receiving algae supplementation exhibited noticeable differences in both the number and intensity of protein bands compared to the control group. Significant changes were observed in protein bands corresponding to molecular weights ranging from approximately (20–60 kDa and 120–150 kDa) across serum, liver and spleen samples, indicating that algal supplementation influenced protein expression profiles in these tissues.

**Table 6.** Dendrogram cluster analysis of protein expression patterns in serum (A), liver (B), and spleen (C) tissues of rabbits fed diets supplemented with two levels of *A. platensis* and *C. vulgaris*.

Band	Spleen					Liver					Serum				
	Control	Ap1	Ap2	Cv1	Cv2	Control	Ap1	Ap2	Cv1	Cv2	Control	Ap1	Ap2	Cv1	Cv2
1	150			150		–	170	170	–	170	–	–	200	–	–
2	130		120	120	120	–	150	150	–	150	–	–	120	–	120
3	90	90	90	90	90	–	120	120	–	120	110	110	–	–	–
4	80	80	70	80	85	110	110	110	–	110	–	–	100	–	–
5	60	60	60	70	80	95	95	95	95	–	85	85	85	85	85
6	50	55		60	75	85	85	85	85	85	72	70	–	70	70
7	35	32	32	50	70	70	70	70	70	70	–	–	–	–	60
8	28	27	27	32	60	65	65	65	65	65	50	–	50	50	–
9	25	23	23	22	45	55	55	55	55	55	–	24	30	25	27
10	17		17	17	32	50	50	50	50	50	–	15	–	15	15
11	13		13	13	27	47	47	47	47	47	–	10	–	–	–
12					22	43	43	43	43	43					
13					18	40	40	40	40	40					
14					16	37	37	37	37	37					
15						32	32	32	32	32					
16						28	28	28	28	28					
17						20	28	–	–	17					
18						15	15	–	–	–					
19						10	10	10	10	10					

## 4. Discussion

The present study demonstrates that dietary supplementation with *A. platensis* and *C. vulgaris* positively influences growth performance, antioxidant status, cecal fermentation, microbial balance, and immune-related gene expression in growing rabbits. Most physiological and immunological responses were more pronounced at the higher supplementation levels (Ap2 and Cv2), indicating a clear dose-dependent physiological response. The improved growth performance observed in algae-supplemented rabbits is consistent with previous reports in rabbits fed dietary supplementation with *Arthrospira* or *Chlorella* [8, 15, 16, 36]. Enhanced body weight gain and FCR may be attributed to the high nutritional value of microalgae, which are rich sources of protein content, essential amino acids, vitamins, minerals, and bioactive compounds [10, 11, 12]. Interestingly, the most efficient FCR was observed at the lower supplementation level, indicating that moderate inclusion may optimize nutrient utilization. In addition, improvements in nutrient utilization and intestinal morphology reported previously [8, 9, 16, 37] support the present findings and suggest that microalgae may enhance digestive efficiency and intestinal absorptive capacity. Oxidative status plays a critical role in maintaining animal health and immune competence [15, 38]. In the current study, the reduction in serum malondialdehyde (MDA) and hydrogen peroxide (H₂O₂) levels in algae-fed rabbits indicates a lower oxidative burden, which agrees with previous studies demonstrating enhanced antioxidant defenses following microalgae supplementation [38, 39, 40, 41, 42]. These effects are biologically plausible given the abundance of antioxidant compounds in *Arthrospira* and *Chlorella*, such as phenolics, flavonoids, carotenoids, phycocyanin, tocopherols, and polyunsaturated fatty acids [15, 38, 39].

Although the algal extracts exhibited higher IC₅₀ values than ascorbic acid in the DPPH and ABTS assays, indicating lower *in vitro* antioxidant potency, the significant reduction in oxidative stress markers (MDA and H₂O₂) observed *in vivo* demonstrates effective physiological antioxidant activity. Collectively, these bioactive compounds may act synergistically to scavenge reactive oxygen species and support endogenous antioxidant systems [41, 42]. Cecal fermentation results revealed increased total volatile fatty acids concentrations, particularly acetate and propionate, alongside reduced ammonia-N concentrations in algae-supplemented groups. These findings indicate improved microbial fermentation efficiency and enhanced nitrogen utilization. Microalgae may indirectly support cecal fermentation by promoting beneficial microbial populations rather than serving as direct fermentable substrates [43, 44]. This interpretation aligns with reports indicating that *Chlorella* supplementation enhances fermentative activity and gut health at moderate inclusion levels [17, 18, 45, 46], whereas excessive inclusion may impair fermentation efficiency [46, 47, 48]. The absence of changes in butyric acid concentration suggests that algal supplementation may be selectively enhanced in specific fermentation pathways. The present study provides novel evidence in rabbits, a species for which data on microalgae–microbiota interactions remain limited. The observed reduction in pathogenic bacteria, coupled with an increase in *Lactobacillus* spp., further indicates a favorable modulation of cecal microbiota. These effects may be attributed to the antimicrobial properties of microalgal secondary metabolites, including phenolics, terpenoids, peptides, polysaccharides, and fatty acids [18, 19, 20, 21]. These compounds can disrupt bacterial membrane integrity, inhibit microbial enzymes, and induce oxidative stress in pathogenic cells, ultimately limiting their proliferation [48, 49, 50, 51]. At the same time, suppression of pathogenic microbes may allow beneficial bacteria to predominate, thereby supporting gut health and immune homeostasis [50, 51, 52]. Immunological responses were further evidenced by the upregulation of anti-inflammatory cytokines, particularly *IL-4* and *IL-10*, in serum and in liver and spleen. These cytokines play pivotal roles in regulating inflammatory balance and promoting humoral immunity. The increased expression observed in algae-fed rabbits reflects immunomodulatory effects, including reduced oxidative stress and improved gut microbial balance. Similar immunostimulatory effects of *Arthrospira* and *Chlorella* have been previously reported in rabbits and other animals [52, 53, 54]. The upregulation of *IL-2* further indicates enhanced immune activation, while the modest increase in *TNF-α* expression in liver tissue suggests controlled immune stimulation rather than excessive inflammation. Protein profile analysis using SDS-PAGE revealed differences in band intensity and distribution among treatment groups, particularly in molecular weight ranges associated with immunomodulatory and antioxidant proteins. Although SDS-PAGE does not allow identification of specific proteins, these observed variations support the molecular findings obtained from cytokine gene expression analyses. Dendrogram clustering further revealed treatment-related similarities and differences among the groups, suggesting systemic physiological responses to algal supplementation. Nevertheless, changes in protein profiles should be interpreted cautiously, as they provide only supportive, rather than definitive, evidence of functional protein regulation. Despite the positive results, this study has certain limitations. The feeding period was short, and protein identification was not performed. Future studies involving longer feeding period trials, advanced proteomic analyses, and functional immunoassays might help to further elucidate the mechanisms underlying the observed immunomodulatory effects.

## 5. Conclusions

*Arthrospira platensis* and *Chlorella vulgaris* show strong potential as beneficial feed supplements for growing rabbits. Their dietary inclusion improved growth performance and feed efficiency, reduced oxidative stress, enhanced antioxidant status, and positively influenced cecal fermentation, microbial populations, and immune-related gene expression. These benefits were more pronounced at higher inclusion levels, indicating a dose-dependent response. Overall, both microalgae are promising natural feed additives for improving rabbit health and productivity under practical feeding conditions.

## Data Availability

The data presented in this study are available from the corresponding author upon reasonable request.

## List of abbreviations

Ap, *Arthrospira platensis*; Cv, *Chlorella vulgaris*; FCR, feed conversion ratio; MDA, malondialdehyde; H₂O₂, hydrogen peroxide; NH₃ – N, ammonia nitrogen; *E. coli, Escherichia coli*; TPC, total polyphenolic content; TFC, total flavonoid content; DMSO, dimethyl sulfoxide; NZW, New Zealand White rabbit; PI, performance index; *p*-value, probability value; cDNA, complementary DNA; VFA, volatile fatty acid; MRS, De Man Rogosa-Sharpe; CFU, colony-forming units; SPSS, statistical product and service solutions; ANOVA, analysis of variance; IC₅₀, half-maximal inhibitory concentration; DPPH, 2,2-diphenyl-1-picrylhydrazyl; ABTS, 2,2′-azino-bis(3-ethylbenzothiazoline-6-sulfonic acid); ROS, reactive oxygen species.
